# Development of the lyrics-based deep learning algorithm for identifying alcohol-related words (LYDIA)

**DOI:** 10.1093/alcalc/agad088

**Published:** 2024-01-17

**Authors:** Abraham Albert Bonela, Zhen He, Dan-Anderson Luxford, Benjamin Riordan, Emmanuel Kuntsche

**Affiliations:** Centre for Alcohol Policy Research, La Trobe University, Melbourne, Australia; Department of Computer Science and Information Technology, La Trobe University, Melbourne, Australia; Department of Computer Science and Information Technology, La Trobe University, Melbourne, Australia; Centre for Alcohol Policy Research, La Trobe University, Melbourne, Australia; Centre for Alcohol Policy Research, La Trobe University, Melbourne, Australia; Centre for Alcohol Policy Research, La Trobe University, Melbourne, Australia

**Keywords:** Alcohol exposure, artificial intelligence, song lyrics, music

## Abstract

**Background:**

Music is an integral part of our lives and is often played in public places like restaurants. People exposed to music that contained alcohol-related lyrics in a bar scenario consumed significantly more alcohol than those exposed to music with less alcohol-related lyrics. Existing methods to quantify alcohol exposure in song lyrics have used manual annotation that is burdensome and time intensive. In this paper, we aim to build a deep learning algorithm (LYDIA) that can automatically detect and identify alcohol exposure and its context in song lyrics.

**Methods:**

We identified 673 potentially alcohol-related words including brand names, urban slang, and beverage names. We collected all the lyrics from the Billboard’s top-100 songs from 1959 to 2020 (N = 6110). We developed an annotation tool to annotate both the alcohol-relation of the word (alcohol, non-alcohol, or unsure) and the context (positive, negative, or neutral) of the word in the song lyrics.

**Results:**

LYDIA achieved an accuracy of 86.6% in identifying the alcohol-relation of the word, and 72.9% in identifying its context. LYDIA can distinguish with an accuracy of 97.24% between the words that have positive and negative relation to alcohol; and with an accuracy of 98.37% between the positive and negative context.

**Conclusion:**

LYDIA can automatically identify alcohol exposure and its context in song lyrics, which will allow for the swift analysis of future lyrics and can be used to help raise awareness about the amount of alcohol in music.

**Highlights**

- Developed a deep learning algorithm (LYDIA) to identify alcohol words in songs.
- LYDIA achieved an accuracy of 86.6% in identifying alcohol-relation of the words.
- LYDIA’s accuracy in identifying positive, negative, or neutral context was 72.9%.
- LYDIA can automatically provide evidence of alcohol in millions of songs.
- This can raise awareness of harms of listening to songs with alcohol words.

## Introduction

Music is one of the most integral parts of our lives and it is omnipresent in both physical and digital environments. For example, music is frequently played in public places (such as restaurants, bars, pubs, at sports games) and on radios, televisions, mobile phone applications (like Spotify, Apple Music), and websites like YouTube. Music serves an important role in socialization in children and adolescents (Communications and Media 2009) and people use music to regulate their mood, to relax, to celebrate, and to express joy. Although music is an important and positive feature of our lives, research also indicates that popular music can shape the attitudes and behaviour of youth regarding alcohol use (Herd 2014).

To date, one experimental study provided evidence that exposure to alcohol-related words in song lyrics can affect alcohol consumption (Engels et al. 2011). The authors manipulated the playlists (by creating a list of songs with alcohol-related words and another list of songs without any alcohol-related words) played in a bar and found that patrons who were exposed to alcohol-containing song lyrics consumed more alcohol. This is an important finding because increased alcohol consumption can lead to both intentional and unintentional injuries including road accidents, violence, and suicide ((WHO) 2021). Given that alcohol exposure in songs may lead to increased alcohol use, understanding how prevalent alcohol is in popular music and understanding how it is portrayed may have implications for prevention and intervention.

Research to date suggests that alcohol is common in popular music. For example, in a content analysis, where authors read and manually annotated lyrics from 793 of the most popular songs between 2005 and 2007, 21.3% of the popular music between had explicit references to alcohol (Primack et al. 2012). Additionally, of the songs that included a reference to alcohol, a quarter contained an alcohol brand name (Primack et al. 2012). Other content analyses have found similar results, concluding that alcohol is common in popular music and increased since 1960 (Herd 2005, Hall et al. 2013, Herd 2014, Pettigrew et al. 2018) and alcohol-related brands are mentioned often in popular music (Primack et al. 2012). Additionally, research has also focused on the emotional context of alcohol (also referred to in the literature as sentiment or valence), finding that most references to alcohol are positive (Christenson et al. 2012, Hall et al. 2013). Understanding the emotional context of alcohol references (e.g. whether it is positive, negative, or neutral) may be particularly important for better understanding its impact on alcohol use behaviours and cognitions. The majority of theories that aim to understand the link between alcohol exposure and media and alcohol use (e.g. social learning theory, social cognitive theory) posit that when alcohol content in media is associated with positive contexts (e.g. relaxation, enjoyment), this influences social norms and alcohol expectancies (e.g. everyone drinks/when people drink, good things happen), which in turn, influence earlier alcohol initiation and increased use (Maisto et al. 1999, Schunk 2012, Davis et al. 2019).

Although the current studies that evaluated alcohol in music are important, they were obtained using content analysis, which is extremely burdensome as it requires researchers to manually read song lyrics and identify the alcohol-related words (including brand names, beverage names, and slangs). This means that, using these existing content analyses methods, whenever researchers would like to extract alcohol-related information in new song, the lyrics have to be manually annotated to identify alcohol-related words which is extremely burdensome and time intensive. To circumvent this, researchers have often only manually annotated a subset of available songs (e.g. top songs in Billboard magazine between 2005–2009). With the release of new songs each week and the millions of songs available today (particularly with the growing popularity of streaming services such as Spotify with 80 million tracks (songs and instrumental music) and Apple Music with 100 million songs at any given time), it is impossible to provide evidence on the proportion of alcohol in these songs using content analysis methodology.

Although intensive, content analyses have been necessary because simply using pattern or word matching to count the number of potential alcohol-related words may not yield accurate results. In contrast to words with only one meaning that is clearly alcohol related (e.g. ‘beer’), other words can have either an alcohol-related or non-alcohol-related meaning. For example, a word like ‘bar’ can: 1) be a place where people gather socially and often drink alcohol; 2) be a rod (iron bar) or 3) refer to restricting someone from something (‘to bar from’). To understand the meaning of words and their potential relation with alcohol, it is essential to understand the context of the lyrics surrounding the words, i.e. at least the previous line and the next line of the line in which the word occurs. Similarly, it is difficult to often estimate the context (e.g. positive/negative) of an alcohol reference using word searches. Although identifying context is extremely common and pre-trained algorithms exist (e.g. LIWC, NRC), they are often unfit for studying alcohol use because they often assume that alcohol use and consequences are negative. For example, using a bag of words approach, the term ‘drunk’ or ‘blackout’ are often seen as negative and using this approach would lead to the conclusion that the reference is negative. However, alcohol references in media are more likely to be positive than negative. For example, in studies that analyse social media posts that references alcohol use or alcohol-related blackout (considered a very negative consequence), the vast majority of references were positive (Cavazos-Rehg et al. 2015). Humans are adept at doing such complex tasks like natural language processing on small-scale data; however, it demands lot of time and effort for us to manually process and annotate large and growing amounts of data (e.g. new songs that are released daily). Hence, it is important to develop research methods that can automatically identify and quantify alcohol-related words (based on the meaning and context of the words) from the multitude of songs.

Fortunately, the advancements in deep learning, a sub-field of artificial intelligence, have produced unprecedented results in natural language processing tasks like text classification, contextual analysis, language understanding, and translation (Wolf et al. 2020). This is particularly the case with the implementation of transfer learning using transformer language models, which were trained on huge corpuses. These deep learning algorithms can perform tasks like text and word classification quickly and efficiently even on large-scale data, addressing the limitations of existing research methods such as content analysis. In this paper, we aim to develop Lyrics based Deep learning Algorithm (LYDIA) to automatically determine alcohol-related words in song lyrics as well as the context (positive, negative, or neutral) in which these words appear in the song lyrics.

## Materials and methods

### Data collection

We collected lyrics of every song that has ever been in the Billboard Top 100 list between the years 1959 and 2020. The Billboard Top 100 (https://www.billboard.com/charts/hot-100/) is a ranking of the top singles in the United States published by the Billboard magazine. Ranks are based on a single’s physical and digital sales, the amount of airplay it receives, and amount it is streamed.

Our data collection involved two steps. First, we used Wikipedia to identify the list of every song title and artist name of Billboard’s top 100 songs from the year 1959 to 2020 and manually scraped these lists. Second, we downloaded the lyrics of those songs using Lyrics Genius Application Programming Interface (https://pypi.org/project/lyricsgenius/). From this lyric dataset, we excluded instrumental songs that included no lyrics (e.g. Sandstorm by Darude). In addition, we also excluded songs that were entirely in non-English language (e.g. Despacito by Luis Fonsi), but we retained songs where some of the lyrics were in a language other than English (e.g. Macarena by Los del Río). In total, we collected 6110 song lyrics from the Billboard’s top-100 songs from 1959 to 2020 and out of which 2024 (33.1%) song lyrics contain at least one potentially alcohol-related word (such as a beverage name like ‘beer’; and slang words like ‘wasted’; for more examples of alcohol-related words see supplementary material). From the 2024 songs, in total 5187 lines, containing potentially alcohol-related words, were annotated by the annotators.

To identify alcohol, we compiled a list of 673 alcohol-related words—including brand names, urban slang, beverage names etc.—from various sources including previous scientific research concerning alcohol-related words in music (Christenson et al. 2012, Siegel et al. 2013), popular culture (Levine 1981), and popular media (Litt et al. 2018, Riordan et al. 2021); Wikipedia lists (e.g. top brands, beverage lists, location lists, intoxication references); lists from the World Health Organisation (WHO 1994), and words used in alcohol-related harm scales (e.g. the Yong Adult Alcohol Consequences Questionnaire; (Kahler et al. 2005)). The supplementary materials contain the final full list of alcohol-related words and their corresponding source.

### Data pre-processing

Although, in theory, finding an alcohol-related word in a song lyric may sound straight forward by considering alcohol-related words and lyrics as a string of characters, in practice it leads to unexpected mismatches such as indicating that the alcohol-related word ‘bar’ is found in ‘Barcelona’. Hence, to avoid these kinds of mismatches, first each word in our dataset and alcohol-related word in our list is transformed into tokens and their corresponding token indices (numerical representation of each token in the vocabulary list) and later the word token indices were used to identify the occurrences of these alcohol-related words in the lyrics by finding the alcohol-related word token indices in each line of the song lyric token indices.

### Dataset preparation and annotation

**Figure 1:**
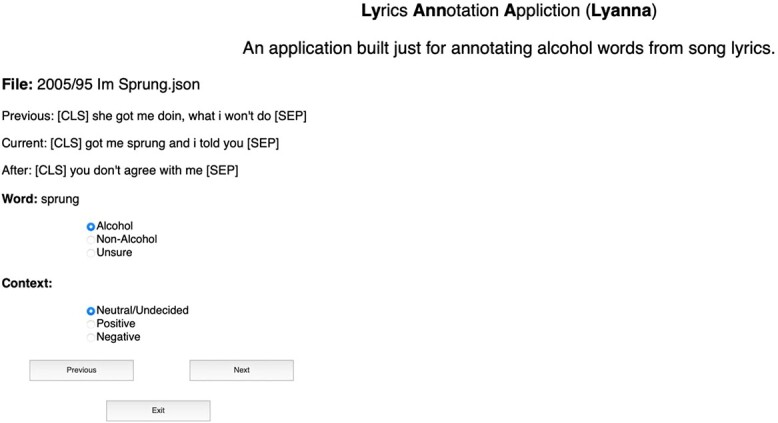
User Interface of the web application created for annotating each line of the song lyrics that contain a word from the alcohol-related words list (see supplementary materials). Note: The words [CLS] and [SEP] indicates the start and end of a line in the song lyrics.

Once we obtained all the potential alcohol-related word occurrences in the lyrics, we annotated each alcohol reference using a custom developed web application called LYANNA (**Ly**rics **Ann**otation **A**pplication as shown in the Fig. 1). Since there is a possibility that more than one alcohol-related word can occur in a line, prior to annotation, we extracted each occurrence of the 673 alcohol-related words from the song lyrics and indexed them into a dataset of 5187 lines (instances). LYANNA handled all these considerations in the background and presented each of those 5187 lines one at a time for annotators to annotate the data. For annotation, LYANNA displays the potentially alcohol-related word and the line of the lyrics that contains the word and the previous and next lines of the current line.

For annotation, the authors (AB, EK, DAL, and BR) met to discuss and define how the data should be annotated. Consequently, we annotated the alcohol-relation of the word (alcohol-related or not) and its context (positive, negative, neural) of all the occurrences of the potentially alcohol-related words in the dataset. For the context, we annotated whether the word is alcohol-related (e.g. *‘I woke up this morning and I got myself a beer’*), non-alcohol related (e.g. *‘every rose has its thorn’;* note the keyword in this instance is rose or rosé), or neutral / mixed (e.g. *‘let’s party’*). In contrast to words like ‘beer’, which were almost always alcohol-related, words like ‘party’ were considered ambiguous if no other context from the line before or line after included a reference to alcohol (e.g. ‘it’s going to be a good night, let’s party, I got on my dancing shoes’). For the context in which the alcohol-related word appeared, we determined whether the context of the line the alcohol-related word appeared in was predominantly positive, negative, or neutral / mixed. For positive, we considered the context positive if the overall line was happy, cool, hopeful (e.g. ‘what a great night let’s get some drinks’), negative if the line was sad, conveyed anxiety (e.g. ‘what a terrible day, I need a drink’), and neutral if we could not discern any emotional context or if it was mixed (e.g. ‘sometimes I will drink’; ‘I’m sad, I’m happy, I drink a beer’). See supplementary material for additional examples. Two alcohol researchers with a background in music and experience with media data (DAL, BR) then annotated all the data. During the annotation process, the two researchers discussed the annotation together, annotated the first 500 songs together, and annotated a substantial number of posts on a zoom call so they could discuss any confusing or difficult examples (see supplementary material for additional information about the annotators and annotation process).

**Figure 2:**
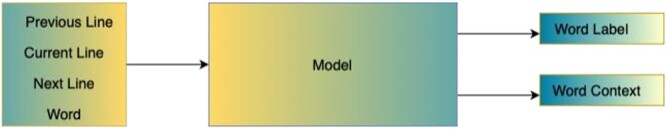
Development of the deep learning algorithm LYDIA. **Note:** The tokenization for previous line, current line, next line is done at sentence level, whereas for word at a word level.

Following annotation, according to standard deep learning algorithm methodology (Kuntsche et al. 2020, Bonela et al. 2022), we split the dataset into training, validation, and test datasets in a ratio of 70%, 10% and 20%, respectively. It is important to note that for each example in the training dataset, current line, previous line, next line, and word were used as input features to the model (Fig. 2). Tokenization for lines was done at sentence level, and tokenization for words was done at word level. All the token indices, per each instance (example) in the dataset, were concatenated before feeding them as inputs to the algorithm. The model outputs the word label and word context.

### Development of the deep learning algorithm (LYDIA)

A single deep learning algorithm, LYDIA, was designed to perform two tasks: 1. Accurately identify alcohol-relation of words (alcohol, non-alcohol, or unsure); 2. Accurately identify the context of the word (positive, negative, or neutral). This is achieved by appending an additional linear layer to the Bert transformer model architecture (Devlin et al. 2018) to predict 6 labels—3 labels (alcohol, non-alcohol, or unsure) for predicting alcohol-relation of the words and 3 labels (positive, negative, or neutral) for predicting the context of the word (see Fig. 2).

**Figure 3:**
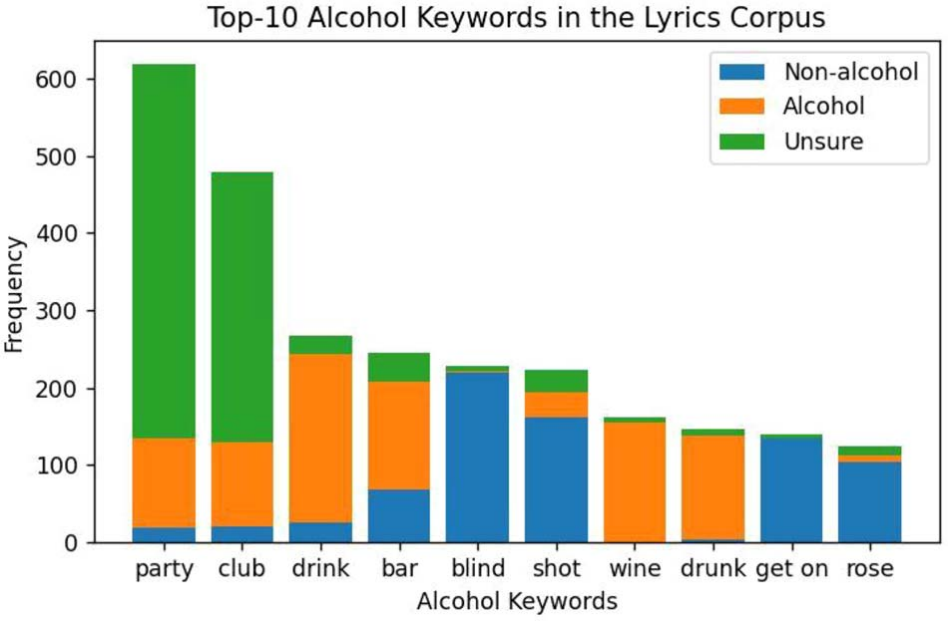
Frequency of the top-10 potentially alcohol-related words that occurred in the lyrics of Billboard’s top 100 songs from the year 1959 to 2020.

Using our training dataset, we finetuned all the parameters of a pretrained Bert model (Devlin et al. 2018) that was initially trained on the *English Wikipedia (2500 million words from text passages)* and the *Toronto book corpus (800 million words from 11,038 unpublished books from 16 different genres).* The advantage of finetuning a pretrained model over training a model exclusively on our dataset is to make use of the existing knowledge learned about the context and connotations of the words from larger corpuses. We trained our algorithm for 20 epochs using the AdamW optimizer (Loshchilov and Hutter 2018) with an initial learning rate of 0.00001. Two cross entropy loss functions (Bridle 1990) were used, one for each task (task-1: alcohol-relation of the word and; task-2: context of the word). The validation dataset was used to find the best model based on the identification of alcohol-relation of the word (task-1) accuracy. Finally, we used the test dataset to evaluate the model performance.

To find the best algorithm that fits the data, in addition to training the algorithm using the Bert (base) transformer model architecture (Devlin et al. 2018), we also trained multiple algorithms using different transformer model architectures—Roberta (base model) (Liu et al. 2019), Albert (base model) (Lan et al. 2019), and Distilbert (base model) (Sanh et al. 2019)—with various initial learning rates and optimizers. It is important to note that for implementing our algorithm training and evaluation, we used the Hugging Face library (https://huggingface.co) which is a natural language processing library that hosts state-of-the-art pretrained models that can be downloaded by anyone to develop their own algorithms for their custom applications.

### Analyses

After providing descriptive statistics about the alcohol-related words found and their context, we evaluated the performance of LYDIA by computing the accuracy, F1-score, and AUC values for both tasks—identifying the alcohol-relation of the word and the context of the word. We computed a confusion matrix, a two-dimensional table comparing the annotator labels with algorithm predictions, to examine which pairs of classes the algorithm was most confused with. We also computed the Cohen’s kappa coefficient as inter-rater reliability between LYDIA and the annotators. Cohen’s kappa values between 0.41–0.60, between 0.61–0.80, and between 0.81–1 represent moderate, substantial, and almost perfect agreement, respectively (Landis and Koch 1977).

## Results

Out of the list of 673 alcohol-related terms, only 236 (35.1%) occurred at least once in the Billboard’s top-100 songs from 1959 to 2020. As shown in the Fig. 3, we found that the word ‘party’ is the most common potentially alcohol-related word in the Billboard’s top-100 songs from 1959 to 2020. Out of the 6110 song lyrics, one third (2024, 33.1%) contained at least one of the potentially alcohol-related words.

LYDIA achieved an accuracy of 86.6% in identifying the alcohol-relation of the word (alcohol, non-alcohol, or unsure; task-1 in Table 1) and 72.9% in identifying the context of the word (positive, negative, or neutral; task-2 in Table 1). According to Table 2, for task-1, LYDIA achieved F1-score and AUC of 85.9% and 94.5%, respectively; for task-2, LYDIA achieved F1-score and AUC of 70.6% and 85.2%, respectively. The results indicate that the finetuned Bert model architecture outperformed the other model architectures in predicting alcohol-related words and their context.

**Table 1:** Performance statistics of multiple algorithms trained on the song lyrics dataset.

	Word Connotation Accuracy			Line Context Accuracy		
Model Architecture	Training	Validation	Test (Task-1)	Training	Validation	Test (Task-2)
Roberta	87.2%	80.7%	82.5%	68.3%	61.7%	63.1%
DistilBert	92.6%	85.4%	85.7%	85.3%	69.0%	70.9%
Albert	93.7%	83.8%	84.5%	87.0%	66.5%	68.2%
**Bert (LYDIA)**	93.9%	86.3%	**86.6%**	89.0%	69.6%	**72.9%**

**Table 2:** Performance statistics of multiple algorithms on the test dataset.

Model Architecture	Accuracy		F1-Score		AUC	
	Word Label	Word Context	Word Label	Word Context	Word Label	Word Context
Roberta	82.5%	63.1%	81.5%	50.7%	92.6%	73.4%
Distilbert	85.7%	70.9%	85.2%	66.5%	**94.5%**	83.0%
Albert	84.5%	68.2%	83.5%	62.8%	93.2%	81.3%
**Bert (LYDIA)**	**86.6%**	**72.9%**	**85.9%**	**70.6%**	**94.5%**	**85.2%**

**Note:** Bold values indicate the best model results for each task.

**Figure 4:**
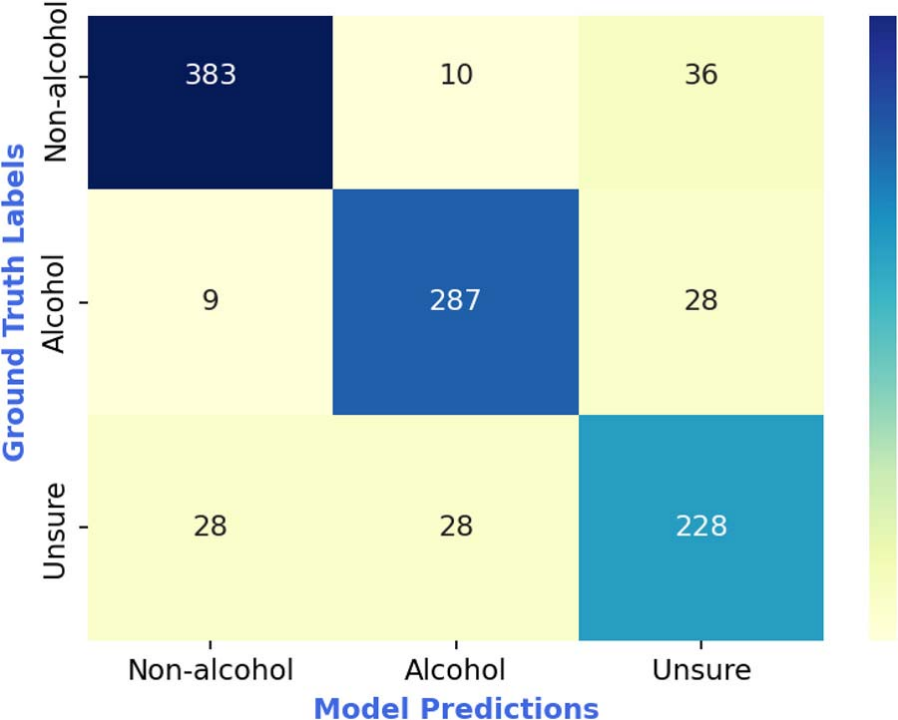
*Confusion matrix of our model (LYDIA) for task-1 (i.e. identifying alcohol-relation of the words).*  **Note:** In the above figure, ground truth labels represent the annotator labels and model predictions represent LYDIA predictions.

The confusion matrix (Fig. 4) for task-1 indicates that misclassification occurred most frequently between the non-alcohol related and unsure categories, compared to the alcohol-related and non-alcohol-related categories. LYDIA can clearly distinguish with an accuracy of 97.2% (= (383 + 287) / (383 + 287 + 10 + 9)) between the words that have positive alcohol-relation and negative alcohol-relation; however, it also indicates that LYDIA is most confused with an error rate of 9.5% (= (36 + 28) / (36 + 28 + 228 + 383)) between words with non-alcohol related or unsure category.

**Figure 5:**
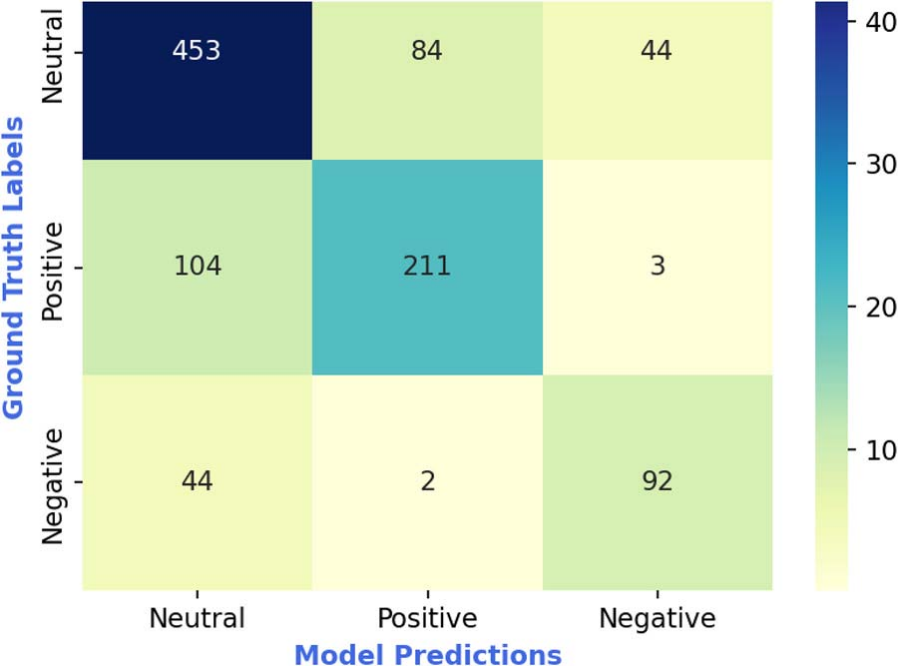
*Confusion matrix of our model (LYDIA) for task-2 (i.e. identifying context of the words).*  **Note:** In the above figure, ground truth labels represent the annotator labels and model predictions represent LYDIA predictions.

Similarly, the confusion matrix of LYDIA computed on task-2 (shown in Fig. 5) indicates that LYDIA is most confused between words with positive context and neutral context, compared to positive context and negative context. LYDIA can clearly distinguish with an accuracy of 98.4% (= (211 + 92) / (211 + 92 + 3 + 2)) between the positive context and negative context; however, it also indicates that LYDIA is relatively highly confused with an error rate of 22.1% (= (84 + 104) / (84 + 104 + 453 + 211)) between the positive context and the neutral context.

Interrater reliability between LYDIA’s predictions and the annotator labels is 0.796 (Cohen’s kappa score) for word label connotation (task-1) suggesting a substantial agreement between annotators and LYDIA and 0.524 for line context (task-2) suggesting a moderate agreement between annotators and LYDIA.

## Discussion

In this paper, we developed LYDIA (a fine-tuned version of BERT model), a deep learning algorithm that can automatically identify alcohol-related words (task-1) and their context (positive, negative, or neutral; task-2) in song lyrics. We found that LYDIA achieved an accuracy of 86.6% and 72.9% for identifying alcohol (task 1) and context (task 2), respectively. The accuracies achieved by LYDIA are 2.6 and 2.2 times higher for task-1 and task-2, respectively, than random chance (that would predict 1 correct answer out of 3; 33.3%). It is also very high considering the complexity of the tasks and the circumstance that the annotators (BR and DAL, who are native English speakers and trained alcohol researchers) found it difficult to decide whether a word such as party or club is alcohol-related or not (see Fig. 1) and occurs in a positive or negative context.

Pertaining to task-1, which is identifying the alcohol-relation of the word, LYDIA was most confused between non-alcohol related and unsure categories, in comparison to alcohol-related and non-alcohol related categories. This could be because of the following reasons. First, the annotators took a conservative approach in labelling the alcohol-related terms as either ‘alcohol’ or ‘unsure’, such that, if terms like ‘club’ or ‘party’ appeared with no reference to alcohol, ‘unsure’ was selected, which most likely generated confusion for the algorithm. In other words, instances of the word ‘club’ may have been assigned ‘unsure’ when alcohol-related, and vice versa. Second, LYDIA appeared to struggle with colloquial and other words which a) appeared infrequently in the dataset, and/or b) are rarely used in the context of alcohol use (e.g. ‘lit’ which can mean drunk/intoxicated but is much more often used for lighting a cigarette, etc.). Finally, and conversely, there were instances where words that often refer to alcohol (e.g. ‘bar’) were misattributed by LYDIA as alcohol-related when used in a non-alcohol-related context (e.g. ‘*Splashing through the sand bar*’). This bias is probably due to scarcity of negative examples in the training dataset for LYDIA to learn when words frequently used in relation to alcohol occur in a non-alcohol related context.

Pertaining to task-2, which is identifying the word context, LYDIA was mainly confused between the positive context and the neutral context in comparison to positive and negative context. This could be for the following reasons. First, in some instances, it was not easy – even for trained annotators – to decide whether the context was positive or neutral, and this difficulty was likely reflected in LYDIA’s performance. This is likely due to the fact that some of the lines were very short. For instance, in several circumstances either “positive” or “neutral” may be an appropriate response. For example, the song lines “*Life is one big party when you’re still young; But who’s gonna have your back when it’s all done?*” could be interpreted as “positive” or “neutral”. As above, LYDIA may have struggled with colloquialisms (e.g. *“Last thing I remember is your beautiful body grindin’ up in the club”*) and other subtle expressions of positive context such as the relief of something negative such as in *“gone are the dark clouds that had me blind”*. Similarly, in some instances, there was mixed context within a song (e.g. one line expressed pain, another relief or joy) which created confusion for the annotators, and therefore also likely affected LYDIA’s performance.

*Limitations.*

One of the main limitations of LYDIA is that it is trained only to identify a subset of alcohol-related words (i.e. only 236 of the 673 alcohol-related words identified (35.1%), see supplementary materials) that occurred at least once in the Billboard’s Top-100 songs in the past 60 years. This is a limitation because if in future applications LYDIA encounters one of the other alcohol-related terms from the list, on which LYDIA was not trained, it may not produce accurate results. Future studies should overcome this limitation by training LYDIA or similar algorithms using datasets that includes a more comprehensive set of alcohol-related words and a greater number of examples in their training data. Additionally, the classification of word context was difficult for the annotators, e.g. when the song lyrics were too short to extract context. Furthermore, the annotated context may have been bias if annotators had knowledge of the song and knew the lyrics or the songs overall subject matter. Furthermore, word context was limited to three discrete categories (i.e. positive, negative, neutral / mixed). This limited the potential to identify instances of mixed emotional context (e.g. both positive and negative) from instances of neutral context (i.e. neither positive nor negative emotions). As such, future work should refine the categories for word context. It is also important to note that there may have been a discrepancy between the emotional context of the alcohol-reference and the overall emotional context of the song and melody. Future research could consider using other tools and resources that estimate the overall emotionality of songs that include an alcohol reference (e.g. Spotify’s API estimates energy, danceability, valence).

*Recommendations and potential health implications.*

One of the main strengths of LYDIA is that it can be applied to automatically quantify alcohol exposure in song lyrics (from the past, present, and future) by identifying the alcohol-related words and their contexts, for example, for compiling alcohol-free songs playlists, i.e. songs that do not include alcohol-related words. Such playlists could help those who aim to reduce their drinking by limiting exposure to alcohol-related cues, which were shown to increase alcohol craving (Field et al. 2008). Hence, alcohol-free song playlists can reduce drinking triggers and temptations (often occurring unconsciously when listening to music) and prevent subsequent alcohol consumption (Engels et al. 2011).

In future work, LYDIA could be extended for music in audio form in order to analyse how often people are exposed to alcohol-related music in their daily life or in different settings. For example, recording music played at a night club, and then using an application like Shazam to identify the song, using Genius’s API, and LYDIA to analyse the lyrics would offer an estimate of alcohol exposure and the context of that exposure from music in a natural setting. Similarly, a number of Ecological Momentary Assessment studies have used applications on mobile devices to intermittently record audio (e.g. Mehl, 2017) and a similar approach could be used to estimate exposure to alcohol-related songs and their context in daily life. Although LYDIA is currently only trained to identify alcohol-related lyrics, other models could be trained to estimate exposure to other health behaviours (e.g. smoking, gambling, sexual content).

## Conclusions

We developed the deep learning algorithm LYDIA that can automatically identify alcohol-related words and the context (positive, negative, or neutral) in which they appear in song lyrics with an accuracy of 86.6% and 72.9%, respectively. LYDIA can potentially be used as a tool for researchers to quantify alcohol exposure in different kinds of existing or future songs and provide information (such as the exposure rate) to the individuals to increase their awareness of alcohol exposure through music.

## Supplementary Material

Supplementary_Material_revised_agad088

## Data Availability

The datasets used in the current study are available from the corresponding author on reasonable request.

## Author Credit Statement

Abraham Albert Bonela: Methodology, Data Curation, Software, Validation, Formal Analysis, Investigation, Writing-Original Draft, Writing-Review & Editing, Visualisation Zhen He: Conceptualization, Methodology, Supervision, Writing-Review & Editing, Formal Analysis, Investigation, Resources, Project Administration Dan-Anderson Luxford: Data Curation, Investigation, Writing-Review & Editing, Formal Analysis, Investigation, Resources Benjamin Riordan: Data Curation, Investigation, Writing-Review & Editing, Formal Analysis, Investigation, Resources Emmanuel Kuntsche: Conceptualization, Supervision, Writing-Review & Editing, Formal Analysis, Investigation, Resources, Project Administration.

## References

[ref1] Bonela AA, He Z, Norman T. et al. Development and validation of the alcoholic beverage identification deep learning algorithm version 2 for quantifying alcohol exposure in electronic images. Alcohol Clin Exp Res 2022;46:1837–45. 10.1111/acer.14925. 36242596 PMC9827927

[ref2] Bridle JS . Probabilistic Interpretation of Feedforward Classification Network Outputs, with Relationships to Statistical Pattern Recognition. Neurocomputing: Springer, 1990.

[ref3] Cavazos-Rehg PA, Krauss MJ, Sowles SJ. et al. “Hey everyone, I’m drunk.” an evaluation of drinking-related twitter chatter. J Stud Alcohol Drugs 2015;76:635–43. 10.15288/jsad.2015.76.635. 26098041 PMC4495081

[ref4] Christenson P, Roberts DF, Bjork N. Booze, drugs, and pop music: trends in substance portrayals in the billboard top 100—1968–2008. Subst Use Misuse 2012;47:121–9. 10.3109/10826084.2012.637433. 22217066

[ref5] COMMUNICATIONS, C. O. & MEDIA . Impact of music, music lyrics, and music videos on children and youth. Pediatrics 2009;124:1488–94. 10.1542/peds.2009-2145. 19841124

[ref6] Davis JP, Pedersen ER, Tucker JS. et al. Long-term associations between substance use-related media exposure, descriptive norms, and alcohol use from adolescence to young adulthood. J Youth Adolesc 2019;48:1311–26. 10.1007/s10964-019-01024-z. 31025156 PMC6816265

[ref7] Devlin J, Chang M-W, Lee K. et al. Bert: pre-training of deep bidirectional transformers for language understanding. 2018; *arXiv preprint arXiv:1810.04805*.

[ref8] Engels RC, Slettenhaar G, Ter Bogt T. et al. Effect of alcohol references in music on alcohol consumption in public drinking places. Am J Addict 2011;20:530–4. 10.1111/j.1521-0391.2011.00182.x. 21999498

[ref9] Field M, Schoenmakers T, Wiers RW. Cognitive processes in alcohol binges: a review and research agenda. Curr Drug Abuse Rev 2008;1:263–79. 10.2174/1874473710801030263. 19630725 PMC3066447

[ref10] Hall PC, West JH, Neeley S. Alcohol, tobacco, and other drug references in lyrics of popular music from 1959 to 2009. Addict Res Theory 2013;21:207–15. 10.3109/16066359.2012.704651.

[ref11] Herd D . Changes in the prevalence of alcohol use in rap song lyrics, 1979–97. Addiction 2005;100:1258–69. 10.1111/j.1360-0443.2005.01192.x. 16128715

[ref12] Herd D . Changes in the prevalence of alcohol in rap music lyrics 1979–2009. Subst Use Misuse 2014;49:333–42. 10.3109/10826084.2013.840003. 24093523

[ref13] Kahler CW, Strong DR, Read JP. Toward efficient and comprehensive measurement of the alcohol problems continuum in college students: the brief young adult alcohol consequences questionnaire. Alcohol Clin Exp Res 2005;29:1180–9. 10.1097/01.ALC.0000171940.95813.A5. 16046873

[ref14] Kuntsche E, Bonela AA, Caluzzi G. et al. How much are we exposed to alcohol in electronic media? Development of the alcoholic beverage identification deep learning algorithm (ABIDLA). Drug Alcohol Depend 2020;208:107841. 10.1016/j.drugalcdep.2020.107841. 31954949

[ref15] Lan Z, Chen M, Goodman S. et al. Albert: a lite bert for self-supervised learning of language representations. 2019; *arXiv preprint arXiv:1909.11942*.

[ref16] Landis JR, Koch GG. An application of hierarchical kappa-type statistics in the assessment of majority agreement among multiple observers. Biometrics 1977;33:363–74. 10.2307/2529786. 884196

[ref17] Levine HG . The vocabulary of drunkenness. J Stud Alcohol 1981;42:1038–51. 10.15288/jsa.1981.42.1038. 7038310

[ref18] Litt DM, Lewis MA, Spiro ES. et al. # drunktwitter: examining the relations between alcohol-related twitter content and alcohol willingness and use among underage young adults. Drug Alcohol Depend 2018;193:75–82. 10.1016/j.drugalcdep.2018.08.021. 30343237 PMC6239902

[ref19] Liu Y, Ott M, Goyal N. et al. Roberta: a robustly optimized bert pretraining approach. 2019; *arXiv preprint arXiv:1907.11692*.

[ref20] Loshchilov I, Hutter F. Fixing Weight Decay Regularization in Adam, 2018.

[ref21] Maisto SA, Carey KB, Bradizza CM. Social Learning Theory, 1999.

[ref1m] Mehl, MR. The Electronically Activated Recorder (EAR): A Method for the Naturalistic Observation of Daily Social Behavior. Current Directions in Psychological Science, 2017;26(2):184–190. 10.1177/0963721416680611. PMC5434514 28529411

[ref22] Pettigrew S, Henriques I, Farrier K. Trends in substance references in Australian top 20 songs between 1990 and 2015. Drug Alcohol Rev 2018;37:S85–8. 29737614 10.1111/dar.12634

[ref23] Primack BA, Nuzzo E, Rice KR. et al. Alcohol brand appearances in US popular music. Addiction 2012;107:557–66. 10.1111/j.1360-0443.2011.03649.x. 22011113 PMC3273659

[ref24] Riordan BC, Merrill JE, Ward RM. et al. When are alcohol-related blackout tweets written in the United States? Addict Behav 2021;107110. 10.1016/j.addbeh.2021.107110 34530209

[ref25] Sanh V, Debut L, Chaumond J. et al. Distilbert, a distilled version of BERT: smaller, faster, cheaper and lighter. 2019; *arXiv preprint arXiv:1910.01108*.

[ref26] Schunk DH . Social Cognitive Theory, 2012.

[ref27] Siegel M, Johnson RM, Tyagi K. et al. Alcohol brand references in US popular music, 2009–2011. Subst Use Misuse 2013;48:1475–84. 10.3109/10826084.2013.793716. 23971875 PMC3830686

[ref28] Who, W. H. O . Lexicon of Alcohol and Drug Terms. World Health Organization, 1994.

[ref29] (WHO), W. H. O . Alcohol [Online]. Available:, 2021, [Accessed 12/10/2021 2021].https://www.who.int/news-room/fact-sheets/detail/alcohol

[ref30] Wolf, T., Debut, L., Sanh, V.et al. Transformers: State-of-the-art natural language processing. Proceedings of the 2020 Conference on Empirical Methods in Natural Language Processing: System Demonstrations, 2020. 38–45.

