# Supplementary material for: Development of the lyrics-based deep learning algorithm for identifying alcohol-related words (LYDIA)
Source: Alcohol Alcohol. 2024 Jan 17;59(2):agad088. doi: 10.1093/alcalc/agad088 (PMC10794165; doi:10.1093/alcalc/agad088)
Supplement: Supplementary_Material_revised_agad088 [file supplementary_material_revised_agad088.docx]

Supplementary Table 1. Examples of context

| **Song Lyrics Examples** | |
| --- | --- |
| Positive | - Got me wanting to put hickeys all over your chest  Ahh, come on we gon' party tonight   Y'all use mouth to mouth bring the party to life   - So shorty just shake it, make a round of applause   If you outta Hypnotic, 'nother round at the bar  And when we parking lot pimping, they surrounding the car   - Burn bad ganja pon his little rowing boat   Red red wine, I'm gonna hold on to you  Hold on to you cause I know your love true   - Been to Nice and the isle of Greece   When I've sipped champagne on a yacht   - It's only up from here, no downward spiral   And I drink to that  Cheers to the freaking weekend |
| Negative | - Maybe we'll someday grow   'Til then just sit your drunk ass on that fuckin' runway, ho   - Here I am without you   Drink to all that we have lost  Mistakes we have made   - One: Don't pick up the phone   You know he's only callin' 'cause he's drunk and alone  Two: Don't let him in   - I've been drinkin', I've been drinkin'   I get filthy when that liquor get into me  I've been thinkin', I've been thinkin'   - 'Til there’s nothing left standing, nothing left of yesterday Every tear-soaked whiskey memory blown away Blown away |
| Neutral | - In the club like what   We be in the party in the club like what   - This old man and me   We’re at the bar and we  We’re having us some beers   - She was staring at her coffee cup   He was trying to keep his courage up by applying booze  Talk was small when they talked at all, they both knew what they wanted   - Midnight, I'm drunk, I don't give a fuck   Wanna dance, by myself, guess you're outta luck   - Whichever way you wanna run, girl   But let me buy you drinks, better yet, rings  Do it how you want it done, girl |

Coding process and annotator backgrounds:

Regarding training and experience, the annotators were two alcohol researchers both experienced and trained musicians with substantial experience conducting content analyses and analysing alcohol-related content in media. Dr. Riordan completed a minor in music (and took papers on performance, theory, and production) and has conducted numerous content analyses with media analyses. He has published extensively on sentiment analyses of media data, including analysing the sentiment of social media posts about cannabis, alcohol, alcohol-related blackouts, health guidelines, etc.

Anderson-Luxford is a PhD student and a guitarist and celloist who performs regularly in Melbourne (with his band “bin night bandits”). He has written and reviewed a substantial amount of literature on alcohol exposure.

The coding scheme was agreed upon during an in person meeting with AB, EK, DAL, and BR. Although there was some discussion around whether we should code the overall context that the alcohol-related word appeared in (e.g., the line before, line with the reference, and line after were predominantly positive or negative) or whether we should include ‘approach’ as positive, even if the context is negative (e.g., “I had a bad day, so I grabbed a beer, life is bleak” would be coded as negative), we opted to code for the overall context given the implications for theory (as justified above). Thus, words were considered to be in a positive, negative and neutral context when the overall context of the line(s) in which the alcohol-related word appeared conveyed positive or negative valence, respectively. Where there was mixed sentiment, or no particular prevailing sentiment could be inferred from the line(s), the instance was annotated as “neutral”.

Anderson-Luxford and Dr. Riordan then discussed the coding together, coded the first 500 songs together, and coded a substantial amount of posts on a zoom call so they could discuss any confusing or difficult examples. They also met regularly during coding to discuss issues.
